# Kombination aus Dezentrierung und Verkippung der Linse im phaken und pseudophaken Auge – optische Simulation von Defokus, Astigmatismus und Coma

**DOI:** 10.1007/s00347-020-01235-x

**Published:** 2020-09-30

**Authors:** Achim Langenbucher, Pooria Omidi, Timo Eppig, Nóra Szentmáry, Rupert Menapace, Peter Hoffmann

**Affiliations:** 1 Institut für Experimentelle Ophthalmologie, Universität des Saarlandes, Kirrberger Str. 100, Gebäude 22, 66424 Homburg, Deutschland; 2 Dr. Rolf M. Schwiete Zentrum für Limbusstammzellforschung und kongenitale Aniridie, Universität des Saarlandes, Kirrberger Str., Gebäude 22, 66421 Homburg, Deutschland; 3 Klinik für Augenheilkunde, Semmelweis-Universität, Mária u. 39, 1085 Budapest, Ungarn; 4 Universitätsklinik für Augenheilkunde und Optometrie, AKH Wien, Wien, Österreich; 5 Augen- und Laserklinik Castrop-Rauxel, Haus am Münsterplatz, Münsterplatz 7, 44575 Castrop-Rauxel, Deutschland

**Keywords:** Intraokularlinse, Linsenausrichtung, Abbildungsqualität, Modellierung, Strahldurchrechnung, Intraocular lens, Lens alignment, Image performance, Modelling, Raytracing

## Abstract

**Hintergrund und Zielsetzung:**

Der Einfluss von Dezentrierung und Verkippung von Kunstlinsen auf die Abbildungsqualität ist in den vergangenen Jahren ausgiebig in Simulationen wie auch klinischen Studien untersucht worden. Ziel dieser Arbeit ist es, den Einfluss der Dezentrierung und Verkippung auf die Induktion von Defokus, Astigmatismus und Coma im phaken und pseudophaken Auge zu untersuchen.

**Methoden:**

Auf der Basis des Liou-Brennan-Modellauges wurde eine Simulation mit Zemax durchgeführt. Ausgehend von der im Augenmodell beschriebenen Position der Gradientenlinse, wurde nach der Bestimmung der Fokusebene die Linse von −1,0 bis 1,0 mm in Schritten von 0,2 mm horizontal dezentriert und von −10° bis 10° in Schritten von 2° um die vertikale Achse verkippt. Zu jeder der 121 Kombinationen wurde bei einer Pupille von 4 mm der Defokus, der reguläre Astigmatismus in 0/180° sowie die horizontale Coma aus der Wellenfront extrahiert. Analog zum phaken Auge wurde die Gradientenlinse durch ein aberrationskorrigierendes Kunstlinsenmodell ersetzt und die Simulation für das pseudophake Auge wiederholt.

**Ergebnisse:**

Ist die Linse im phaken/pseudophaken Auge entsprechend den Vorgaben des Liou-Brennan-Modellauges positioniert, ergibt die Simulation einen Defokus von 0,026/−0,001 dpt, einen Astigmatismus von −0,045/−0,018 dpt sowie eine Coma von −0,015/0,047 µm. Maximale Werte treten bei einer Dezentrierung von 1,0 mm und einer Verkippung von 10° auf: 1,547/2,982 dpt für den Defokus, 0,971/1,871 dpt für den Astigmatismus sowie 0,441/1,209 µm für die Coma. Maximal negative Werte treten im phaken/pseudophaken Auge auf bei: −0,293/−1,224 dpt für den Defokus, −0,625/−0,663 dpt für den Astigmatismus sowie −0,491/−0,559 µm für die Coma.

**Diskussion:**

In dieser Studie wurde erstmals der Effekt einer Kombination aus horizontaler Dezentrierung der Linse und Verkippung um die Vertikale auf den induzierten Defokus, Astigmatismus sowie die horizontale Coma in einem Simulationsmodell untersucht. Die Ergebnisse können bei der Ursachenforschung helfen, wenn bei dezentrierter oder verkippter Kunstlinse die Zielrefraktion nicht mit der erreichten Refraktion übereinstimmt oder der resultierende Astigmatismus durch den Hornhautastigmatismus alleine nicht erklärbar ist.

## Abstract (en)

**Background and purpose:**

The effect of lens decentration and tilt on retinal image quality has been extensively studied in the past in simulations and clinical studies. The purpose of this study was to analyze the effect of combined lens decentration and tilt on the induction of defocus, astigmatism and coma in phakic and pseudophakic eyes.

**Methods:**

Simulations were performed with Zemax on the Liou-Brennan schematic model eye. Based on the position of the gradient lens the image plane was determined (best focus). The lens was decentered horizontally from −1.0 mm to 1.0 mm in steps of 0.2 mm and tilted with respect to the vertical axis from −10° to 10° in steps of 2° (in total 121 combinations of decentration and tilt). For each combination of decentration and tilt defocus, astigmatism (in 0/180°) and horizontal coma was extracted from wave front error and recorded for a pupil size of 4 mm. After replacement of the gradient lens with an aberration correcting artificial lens implant model with the equatorial plane of the artificial lens aligned to the equatorial plane of the gradient lens, the simulations were repeated for the pseudophakic eye model.

**Results:**

For the lens positioned according to the Liou-Brennan schematic model eye the simulation yielded a defocus of 0.026 dpt/−0.001 dpt, astigmatism of −0.045 dpt/−0.018 dpt, and a coma of −0.015 µm/0.047 µm for phakic/pseudophakic eyes. Maximum values were observed for a horizontal decentration of 1.0 mm and a tilt with respect to the vertical axis of 10° with 1.547 dpt/2.982 dpt for defocus, 0.971 dpt/1.871 dpt for astigmatism, and 0.441 µm/1.209 µm for coma. Maximum negative values occurred in phakic/pseudophakic eyes with −0.293 dpt/−1.224 dpt for defocus, for astigmatism −0.625 dpt/−0.663 dpt and for coma −0.491 µm /−0.559 µm, respectively.

**Conclusion:**

In this simulation study the effect of a combination of lens decentration in horizontal direction and tilt with respect to the vertical axis on defocus, astigmatism and horizontal coma was analyzed. The results may help to describe in clinical routine if with a decentered or tilted artificial lens implant the postoperative refraction does not match the target refraction or the resulting astigmatism after cataract surgery is not fully explained by measurement of corneal astigmatism.

Die Dezentrierung und Verkippung der natürlichen Augenlinse im phaken Auge bzw. der Kunstlinse im pseudophaken Auge nach einer Kataraktoperation konnte für viele Jahre nur abgeschätzt werden [22]. Zur Verfügung standen dafür optische Messverfahren wie Purkinje-Meter oder Scheimpflug-Kameras [4, 6, 16]. Allerdings ist speziell bei Scheimpflug-Kameras das Messfeld in axiale Richtung deutlich begrenzt, da die Einhaltung der Scheimpflug-Bedingung voraussetzt, dass die Bildebene, die Linsenebene sowie die Ebene der Spaltbeleuchtung in eine Linie schneiden. Bei Purkinje-Metern sind dagegen vereinfachende Annahmen über die Grenzflächen der Hornhaut und die axiale Position der Linse und deren Krümmungsradien nötig, um absolute Messgrößen zur Dezentrierung und Verkippung der Augenlinse zu extrahieren.

Mit der neuen Generation der optischen Kohärenztomographie für den vorderen Augenabschnitt hat der Kliniker erstmals die Möglichkeit, in einer Messung mit hoher Auflösung den gesamten vorderen Augenabschnitt zu vermessen [21]. Das Messfenster von lateral deutlich mehr als 12 mm und axial mehr als 10 mm reicht aus, um neben der Hornhaut sowohl die natürliche Augenlinse wie auch die Kunstlinse nach der Kataraktoperation zu vermessen. Zum Teil existieren entsprechende Applikationen oder Messmodalitäten, die speziell für die Auswertung der Dezentrierung und Verkippung der Augenlinse entwickelt wurden [19].

Die Auswirkung der Dezentrierung oder Verkippung von Kunstlinsen wurde in der Literatur hinreichend adressiert. Meist stehen bei diesen Studien das Abbildungsverhalten oder optische Kenngrößen wie die „point spread function“ oder Modulationstransferfunktion beim pseudophaken Auge im Vordergrund [1, 3–5, 7, 9–11, 13, 17, 18, 23]. So wurden vergleichende Untersuchungen an sphärischen wie auch asphärisch aberrationsneutralen und aberrationskorrigierenden Kunstlinsen durchgeführt. Systematische Untersuchungen zu Defokus und Astigmatismus oder der Veränderung der Sehachse sind selten [8, 9, 11, 19].

Betrachtet man moderne schematische Augenmodelle wie das Liou-Brennan-Auge [14], so kann direkt eine Asymmetrie in horizontaler Richtung abgelesen werde. Dadurch, dass die Fovea nach temporal ausweicht, verläuft die Sehachse nicht zentriert durch das Auge. Vielmehr wird von einer horizontalen Verkippung des Strahls von etwa 5° (Winkel alpha bzw. kappa) ausgegangen, wodurch die optischen Elemente Hornhaut und Linse gegenüber der Fixationsachse in horizontaler Richtung dezentriert und um die vertikale Achse verkippt erscheinen. In vertikaler Richtung ist dagegen in derartigen Modellaugen keine Asymmetrie vorgesehen, sodass die Hornhaut und Linse in vertikale Richtung keine Dezentrierung sowie um die horizontale Achse keine Verkippung gegenüber der Sehachse erfahren [2, 14].

Nach einer Kataraktoperation mit Implantation einer Kunstlinse in den Kapselsack richtet sich das Implantat in der Regel so aus, dass sich die Haptikebene der Linse näherungsweise in der Äquatorebene der natürlichen Linse positioniert [20, 21]. Das bedeutet, dass die Kunstlinse in grober Abschätzung eine Dezentrierung und Verkippung zur Fixationsachse aufweist, die vergleichbar der natürlichen Linse ist.

Allerdings wurden nach unserem Kenntnisstand der zu erwartende Defokus, Astigmatismus sowie die Coma am phaken und pseudophaken Auge bei horizontaler Dezentrierung in Kombination mit einer Verkippung um die vertikale Achse nicht systematisch untersucht.

Das Ziel der vorliegenden Arbeit ist es, in einem Simulationsmodell, basierend auf dem schematischen Modellauge nach Liou-Brennan, die natürliche Linse in horizontale Richtung zu dezentrieren sowie um die vertikale Achse zu verkippen und den resultierenden Defokus, Astigmatismus sowie die Coma in der Fokalebene zu ermitteln. Des Weiteren soll die natürliche Linse durch eine aberrationskorrigierende Kunstlinse ersetzt werden, und am pseudophaken Auge sollen ebenfalls der resultierende Defokus, Astigmatismus sowie die Coma bei Dezentrierung der Linse in horizontale Richtung und Verkippung um die vertikale Achse untersucht werden.

## Methoden

Als Ausgangspunkt für die Simulation in ZEMAX wird das schematische Modellauge nach Liou-Brennan herangezogen [14]. Das Auge ist so definiert, dass der Eingangsstrahl in horizontale Richtung um 5° nach nasal geneigt ist und somit die Fixationsachse gegenüber der Symmetrieachse geneigt ist [2, 14, 19]. Für die Blende des optischen Systems wird ein Durchmesser von 4 mm in der Pupillenebene gewählt, sodass auch bei einer Dezentrierung von 1 mm sowie einer Verkippung von 10° kein Strahl außerhalb der pseudophaken Linsenoptik verläuft, wenn man einen typischen Optikdurchmesser von 6 mm annimmt [12]. Die Fovea wird in die Ebene mit dem besten Fokus gelegt. Als Beleuchtung wurde ein monochromatischer (Wellenlänge = 500 nm) kollimierter Eingangsstrahl (entsprechend einem Objekt im Unendlichen) definiert.

Ausgehend von dieser Konstellation, wird die kristalline Linse des Auges in einem Bereich von ±1,0 mm (Schrittweite 0,2 mm) in horizontale Richtung dezentriert sowie in einem Bereich von ±10° (Schrittweite 2°) um die vertikale Achse verkippt. Somit ergeben sich insgesamt 11 × 11 = 121 Szenarien für Kombinationen aus Dezentrierung und Verkippung. Für jedes Szenario wurden der resultierende Astigmatismus, der Defokus sowie die Coma als die wichtigsten optischen Abbildungsfehler bei dezentrierten und verkippten Grenzflächen protokolliert. Die Wellenfrontfehler Defokus und Astigmatismus wurden zur besseren Interpretation in Dioptrien umgerechnet.

In einem zweiten Schritt wurde die natürliche Augenlinse mit Gradientenindex gegen eine aberrationskorrigierende Kunstlinse ausgetauscht (Modell: Z9000 der Fa. Advanced medical Optics, Santa Ana, CA, USA, jetzt Johnson & Johnson Vision, Brechwert 21 dpt, Designdaten wurden aus der Patentschrift entnommen [4]). Die Linse wurde so im Auge positioniert, dass die Haptikebene der Kunstlinse nach der Äquatorebene der natürlichen Linse ausgerichtet wurde. Anschließend wurde die Bildebene so gewählt, dass sie in der Ebene des besten Fokus zu liegen kommt. Ausgehend von dieser Positionierung, wurde die Kunstlinse in einem Bereich von ±1,0 mm (Schrittweite 0,2 mm) horizontal dezentriert sowie in einem Bereich von ±10° (Schrittweite 2°) um die vertikale Achse verkippt. Auch hier wurden insgesamt 121 Kombinationen aus Dezentrierung und Verkippung berücksichtigt. Vergleichbar zum Vorgehen beim phaken Liou-Brennan-Modellauge wurden der Defokus, der resultierende Astigmatismus sowie die Coma protokolliert. Die Wellenfrontfehler Defokus und Astigmatismus wurden zur besseren Interpretation in Dioptrien umgerechnet.

**Figure 1:**
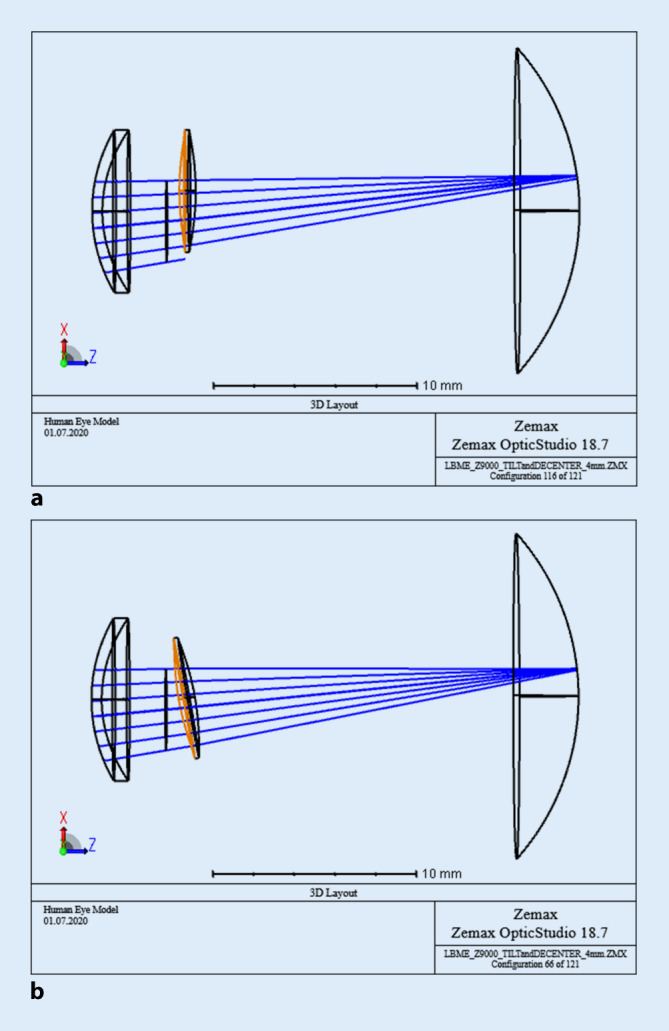

Die Abb. 1 zeigt exemplarisch das pseudophake Augenmodell (nach Ersatz der natürlichen Augenlinse durch eine Kunstlinse) eines rechten Auges von oben. Die Abb. 1a kennzeichnet die Dezentrierung der Linse um +1,0 mm in horizontale Richtung und Abb. 1b die Verkippung um die vertikale Achse um +10°. Die entsprechende Situation für linke Augen ergibt sich durch eine Spiegelung an der vertikalen Achse.

Zur grafischen Aufarbeitung der Ergebnisse wurden Darstellungen gewählt, bei denen, ausgehend von der Positionierung der Augenlinse im Liou-Brennan-Modellauge (entsprechend Dezentrierung und Verkippung gleich null), die Dezentrierung der Linse in horizontale Richtung auf der Abszisse, die Verkippung der Linse um die vertikale Achse auf der Ordinate und die Zielgrößen Defokus, Astigmatismus oder Coma in Farbe kodiert aufgetragen wurden.

## Ergebnisse

Bei der Dezentrierung der Linse in horizontale Richtung sowie Verkippung um die vertikale Achse (Gradientenlinse im phaken Auge oder Kunstlinse im pseudophaken Auge) sind der resultierende Astigmatismus in den schrägen Achsen (45°/135°) sowie die vertikale Coma (90°) gleich null. Somit konnte vereinfachend auf die Darstellung des schrägen Astigmatismus (45° bzw. 135°) sowie der vertikalen Coma verzichtet werden.

Ist die Linse im phaken/pseudophaken Auge entsprechend den Vorgaben im Liou-Brennan-Modellauge positioniert, ergibt sich ein Defokus von 0,026/−0,001 dpt, ein Astigmatismus in 0° von −0,045/−0,018 dpt sowie eine horizontale Coma von −0,015/0,047 µm.

**Figure 2:**
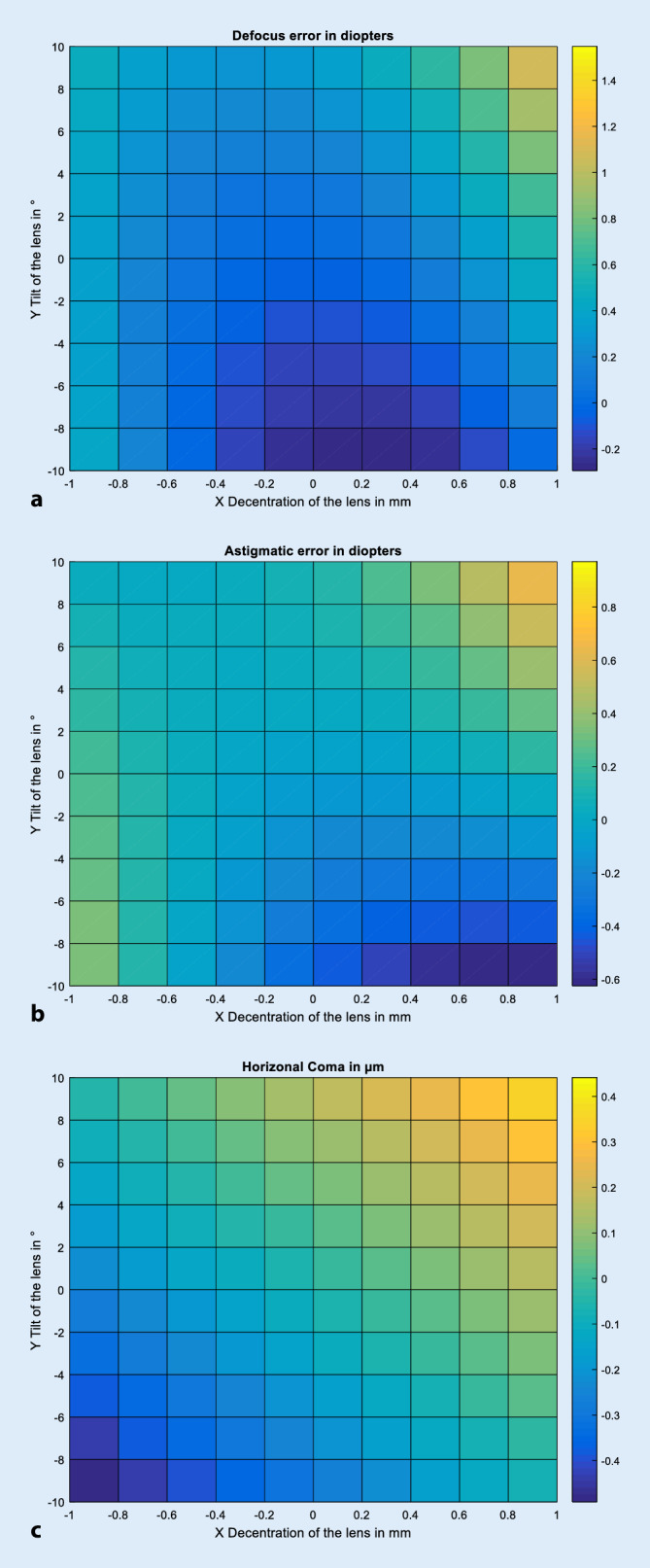

Die Abb. 2 zeigt die Abhängigkeit der Zielgrößen Defokus, Astigmatismus und Coma für verschiedene Kombinationen aus horizontaler Dezentrierung der Linse und Verkippung der Linse um die vertikale Achse für das phake Augenmodell. Aufgrund der Wahl der Bildebene im „best focus“ ist der Defokus ohne Dezentrierung und Verkippung (Dezentrierung und Verkippung der natürlichen Linse entsprechen den Gegebenheiten des Liou-Brennan-Modellauges) sehr gering. Die zugehörigen Skalen sind dem Farbbalken neben der jeweiligen Grafik zu entnehmen. Die Abb. 2a stellt den Defokus für das phake Auge dar. Speziell Kombinationen aus hoher positiver Dezentrierung und hoher positiver Verkippung führen zu einem hohen Defokusfehler (bei 1,0 mm Dezentrierung und 10° Verkippung: 1,547 dpt). Maximal negative Werte traten für eine Dezentrierung von 0,0 mm und eine Verkippung von −10° auf (−0,293 dpt). Die Abb. 2b stellt den Astigmatismus in horizontale Richtung (0°/180°) dar. Hohe positive Dezentrierungen in Kombination mit hoher positiver Verkippung führen zu einem starken Astigmatismus (bei 1,0 mm Dezentrierung und 10° Verkippung: 0,971 dpt). Maximal negative Werte traten für eine Dezentrierung von 0,8 mm und eine Verkippung von −10° auf (−0,625 dpt). Die Abb. 2c stellt die horizontale Coma in Falschfarbenkodierung dar. Die horizontale Coma ist besonders ausgeprägt bei Kombinationen aus deutlicher positiver Dezentrierung und Verkippung der Linse (Dezentrierung 1,0 mm und Verkippung 10°: 0,441 µm). Maximal negative Werte traten für eine Dezentrierung von −1,0 mm und eine Verkippung von −10° auf (−0,491 µm).

**Figure 3:**
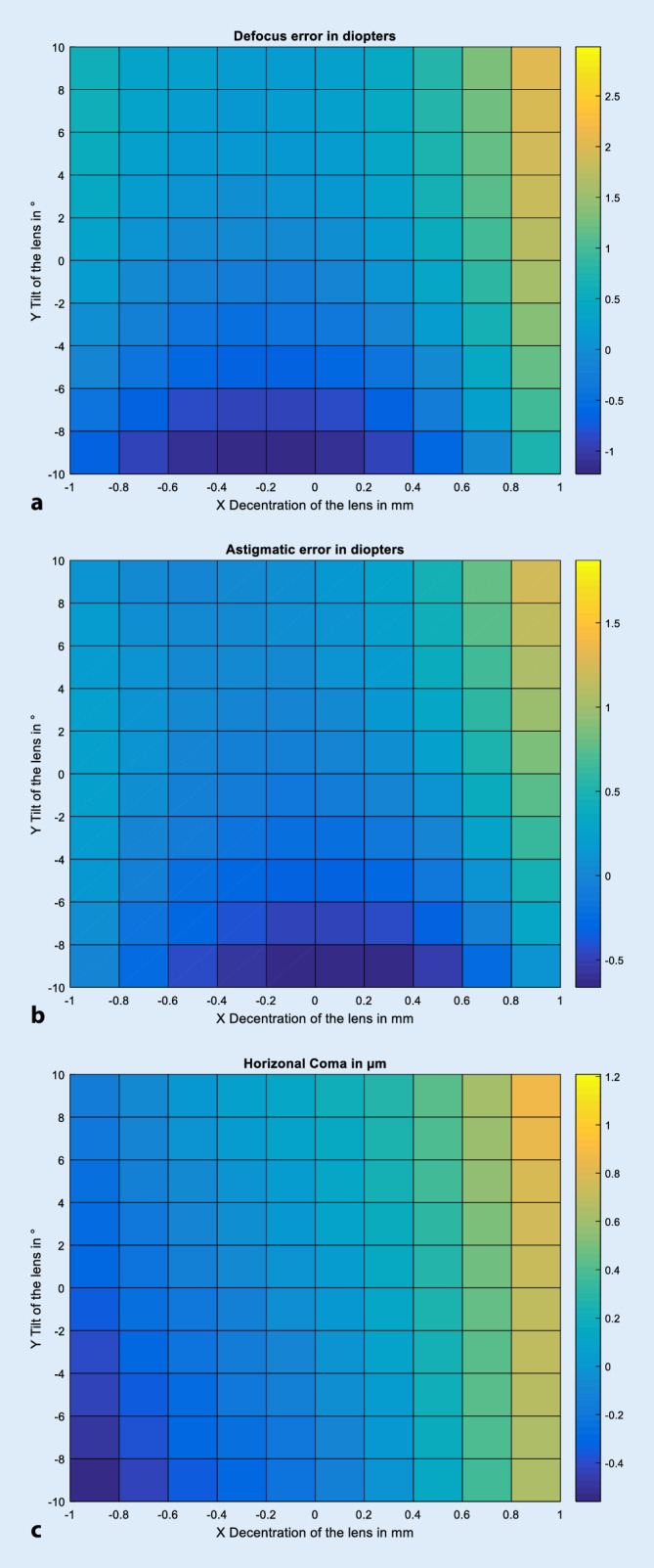

Die Abb. 3 zeigt die Abhängigkeit der Zielgrößen Defokus, Astigmatismus in 0/180° und horizontale Coma für verschiedene Kombinationen aus horizontaler Dezentrierung der Linse und Verkippung der Linse um die vertikale Achse für das pseudophake Augenmodell mit einer aberrationskorrigierenden Z9000-Kunstlinse. Aufgrund der Wahl der Bildebene nach dem „best focus“ ist der Defokus ohne Dezentrierung und Verkippung (die Haptikebene der Kunstlinse stimmt mit der Äquatorebene der Gradientenlinse im phaken Augenmodell überein) sehr gering. Die zugehörigen Skalen sind dem Farbbalken neben der jeweiligen Grafik zu entnehmen. Die Abb. 3a stellt den Defokus im pseudophaken Auge dar. Speziell für eine große positive Dezentrierung und Verkippung der Kunstlinse wird ein deutlicher Defokus beobachtet (Dezentrierung von 1,0 mm und Verkippung von 10°: 2,982 dpt). Maximal negative Werte traten für eine Dezentrierung von 0,2 mm und eine Verkippung von −10° auf (−1,224 dpt). Die Abb. 3b stellt den Astigmatismus in 0°/180° für das pseudophake Auge dar. Speziell für eine große positive Dezentrierung und Verkippung der Kunstlinse wird ein deutlicher Astigmatismus beobachtet (Dezentrierung von 1,0 mm und Verkippung von 10°: 1,871 dpt). Maximal negative Werte traten für eine Dezentrierung von 0,0 mm und eine Verkippung von −10° auf (−0,663 dpt). Die Abb. 3c zeigt die horizontale Coma für das pseudophake Auge in Falschfarbenkodierung. Die horizontale Coma ist besonders ausgeprägt bei Kombinationen aus starker positiver Dezentrierung und Verkippung der Linse (Dezentrierung von 1,0 mm und Verkippung von 10°: 1,209 µm). Maximal negative Werte traten für eine Dezentrierung von −1,0 mm und eine Verkippung von −10° auf (−0,559 µm).

**Figure 4:**
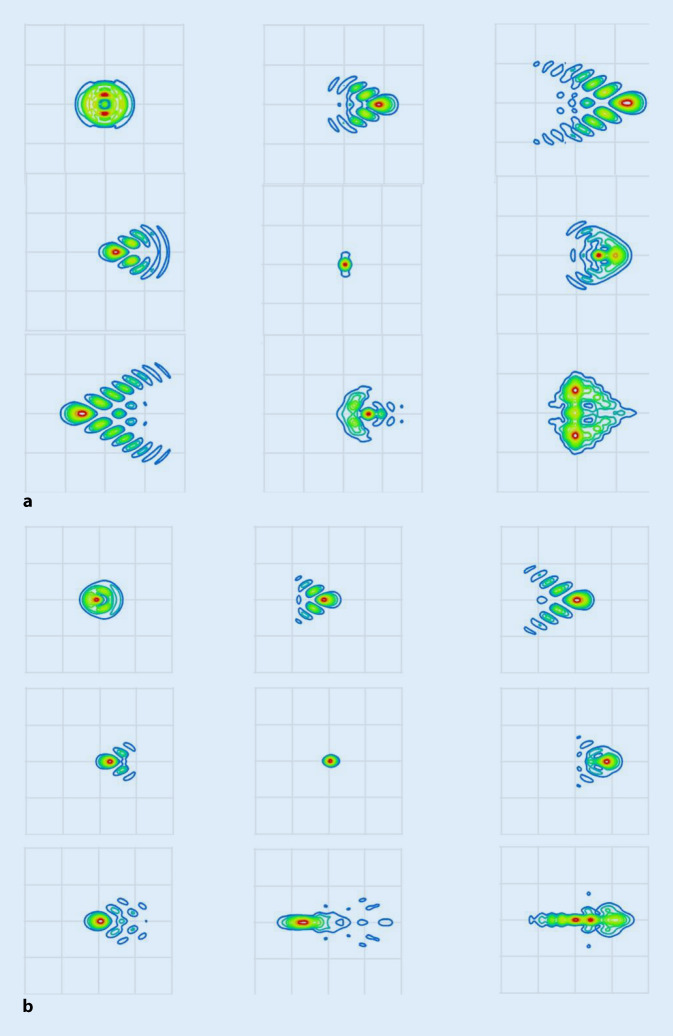

Die Abb. 4 zeigt für das phake (Abb. 4a) und pseudophake (Abb. 4b) Auge exemplarisch für 9 der 121 ausgewählten Szenarien (Kombinationen aus Dezentrierung von 0,6/0,0/0,6 mm und Verkippung von −6/0/6°) die Punktbildverwaschungsfunktion („point spread function“) in der Bildebene. Beide Abbildungen zeigen qualitativ, dass die Abbildungsfehler beim phaken wie auch beim pseudophaken Auge mit Ausnahme der Linsenpositionierung gemäß Liou-Brennan-Augenmodell (jeweils mittleres Bild) und der Kombination aus Dezentrierung von −0,6 mm und Verkippung von −6° durch die horizontale Coma dominiert werden, der Einfluss des Astigmatismus auf die Punktbildverwaschungsfunktion spielt hier eine untergeordnete Rolle.

## Diskussion

Viele schematische Augenmodelle (z. B. Gullstrand, Kooijman, Lotmar etc.) vereinfachen das Auge als ein rotationssymmetrisches optisches System, bei dem sphärische oder asphärische optische Grenzflächen unterschiedliche Medien voneinander trennen [2]. Tatsächlich liegt beim menschlichen Auge aufgrund der exzentrischen Lage der Foveola keine Symmetrie in horizontale Richtung vor. Die Stelle des schärfsten Sehens weicht aus dem hinteren Pol nach temporal aus. Somit verläuft die Sehachse des Auges um etwa 4–7° in horizontale Richtung geneigt [2]. Diese Neigung der Sehachse relativ zur idealisierten optischen Achse wird als Winkel α bezeichnet. Hiervon unterscheidet sich der klinisch verwendete Winkel κ, der die Neigung der Sehachse zur Pupillenachse (Lot auf der Hornhaut, welches das Pupillenzentrum schneidet) angibt und welcher deutlich geringer ausfällt als α. Aufgrund des schrägen Durchlaufs der Sehachse durch die optischen Medien trifft die Sehachse die Hornhaut und Linse nicht senkrecht oder zentriert [21]. Im Liou-Brennan-Modellauge ist dieser Verkippung der Sehachse dadurch Rechnung getragen, dass zwar die optischen Elemente Hornhaut und Augenlinse zentriert zur Symmetrieachse angeordnet sind, jedoch der Eingangsstrahl um 5° in der Horizontalen nach nasal verkippt ist [14].

Ein optisches System mit dezentrierten oder verkippten optischen Elementen weist Abbildungsfehler auf, die in zentrierten Systemen vernachlässigt werden können. Neben dem Astigmatismus schiefer Bündel tritt v. a. ein Coma-Fehler auf, der sich in der Bildebene durch eine Konfiguration vergleichbar einem Kometenschweif äußert. Zusätzlich verschiebt sich der Fokus in axiale Richtung, wodurch sich der Brechwert des optischen Systems verändert. In der Ophthalmologie wird oft unter einem Astigmatismus sowohl der reguläre Anteil, der mit einer zylindrischen Korrektur kompensiert werden kann, wie auch der irreguläre Anteil verstanden, der ausschließlich durch eine individuelle Wellenfrontkorrektur behoben werden kann.

In der vorliegenden Arbeit wurde ein Simulationsansatz gewählt, bei dem, ausgehend von einem modernen nicht zentrierten schematischen Augenmodell (Liou-Brennan [14]), eine Strahldurchrechnung erfolgte. Dabei wurde zunächst im phaken Modellauge die Bildebene („best focus“) ermittelt und anschließend die natürliche Linse (Gradientenlinse) in horizontale Richtung dezentriert und um die vertikale Achse verkippt. Insgesamt wurden jeweils für die Dezentrierung und Verkippung 11 Szenarien durchgerechnet, sodass sich insgesamt in einem Bereich der Dezentrierung von −1,0 bis 1,0 mm und einem Bereich der Verkippung von −10 bis 10° 121 Kombinationen ergaben. Die Bildebene wurde dabei nicht verändert. Aus dem Wellenfrontfehler wurden der Defokusanteil, der Anteil des regulären durch eine zylindrische Korrektur kompensierbaren Astigmatismus (in 0/180°, in den schrägen Achsen ergibt sich bei unserem Ansatz ein Wert identisch 0) sowie die horizontale Coma (vertikale Coma ist aufgrund unseres Ansatzes identisch 0) extrahiert. In einem zweiten Schritt wurde die Gradientenlinse des Liou-Brennan-Augenmodells (stellvertretend für die natürliche Augenlinse) durch eine Kunstlinse ersetzt (Z9000, 21 dpt Brechwert, Designdaten aus der Patentschrift entnommen). Die Linse wurde zunächst so im pseudophaken Modellauge platziert, dass die Haptikebene der Kunstlinse mit der Äquatorebene der natürlichen Linse im phaken Modellauge übereinstimmt [6, 20]. Danach wurde die Bildebene justiert, um eine Abbildung im Fokus zu erreichen („best focus“). Anschließend wurde entsprechend dem Vorgehen beim phaken Modellauge die Kunstlinse im Bereich von −1,0 bis 1,0 mm dezentriert und im Bereich −10° bis 10° verkippt.

Aus den Abb. 2a, b sowie 3a, b kann man direkt ablesen, dass die isolierte Betrachtung von Dezentrierung und Verkippung der Linse eine unrealistische Vereinfachung darstellt und die Kombination aus beidem ggf. die Beeinträchtigung der Abbildungsleistung verstärken oder auch abschwächen kann [4, 5, 15]. Beim phaken wie auch beim pseudophaken Auge verhalten sich der induzierte Defokus sowie der induzierte reguläre Astigmatismus grundsätzlich ähnlich: Für deutlich positive Werte für Dezentrierung und Verkippung treten jeweils der höchste positive Wert im Defokus sowie der höchste positive Wert im regulären Astigmatismus auf (jeweils orangene Bereiche in den Abb. 2a, b und 3a, b). Beim phaken Auge können innerhalb des simulierten Parameterfensters der Dezentrierung/Verkippung rund 1,5 dpt an Defokus bzw. 1,0 dpt an Astigmatismus induziert werden. *Beim pseudophaken Auge liegen die entsprechenden Werte für den Defokus bei maximal 3,0* *dpt bzw. für den regulären Astigmatismus bei maximal 1,9* *dpt, also deutlich höhere Werte im Vergleich zum phaken Auge*. Das Ausmaß des induzierten Defokus, Astigmatismus sowie der Coma beim pseudophaken Auge hängt natürlich deutlich vom Design der Intraokularlinse ab, aus der Literatur ist bekannt, dass sphärische oder aberrationsneutrale Intraokularlinsen deutlich toleranter auf Dezentrierung und Verkippung reagieren als die hier exemplarisch gezeigte aberrationskorrigierende Linse mit einem hohen Korrekturgrad für die sphärische Aberration [4, 5]. Ein sehr viel milderer negativer Defokus sowie Astigmatismus werden dagegen induziert bei geringen Werten der Dezentrierung und deutlich negativen Werten für die Verkippung (blauer Bereich mittig unten in den Abb. 2a, b und 3a, b. Für den Defokus bedeutet ein positiver Wert eine Myopisierung des Auges und ein negativer Wert eine Hyperopisierung des Auges. Für den Astigmatismus bedeuten positive Werte einen induzierten Astigmatismus in 0°, negative Werte dagegen einen Astigmatismus in 90°. Bei der horizontalen Coma ist die Charakteristik beim phaken und pseudophaken Auge unterschiedlich zu Defokus und Astigmatismus. Die Kombination aus starker positiver Dezentrierung und Verkippung liefert auch hier hohe positive Werte für die Coma (0,44 für das phake und 1,21 µm für das pseudophake Auge; orangene Bereiche in Abb. 2c und 3c, rechts oben), jedoch liefert in der Berechnung die Kombination aus starker negativer Dezentrierung und Verkippung einen hohen Wert an negativer Coma von −0,49 µm für das phake und −0,56 µm für das pseudophake Auge (blaue Bereiche in Abb. 2c und 3c, links unten). Zwischen der positiven Coma im Bild rechts oben und der negativen Coma im Bild links unten besteht beim phaken Auge eine Symmetrie, beim pseudophaken Auge nicht.

**Figure 5:**
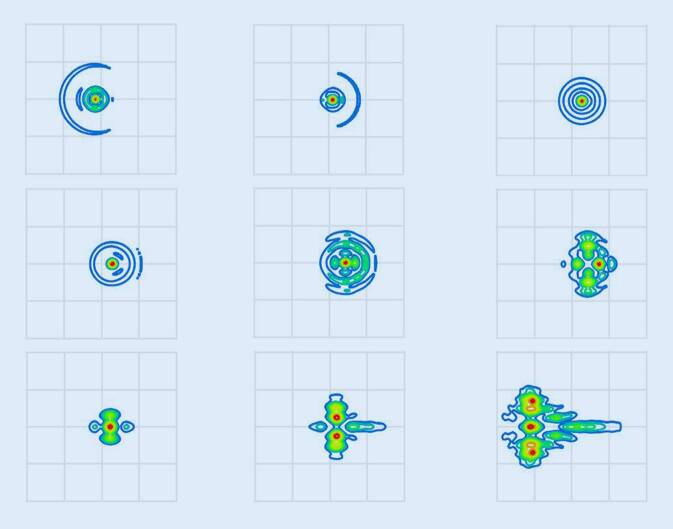

Die Ergebnisse zeigen auf, dass sich die Abbildungsqualität im phaken wie auch im pseudophaken Auge bei Dezentrierung der Linse in horizontale Richtung und Verkippung um die vertikale Achse deutlich verschlechtert. In der vorliegenden Studie wurden ausschließlich horizontale Dezentrierungen und Verkippungen der Linse um die vertikale Achse berücksichtigt, im allgemeinen Fall können natürlich auch vertikale Dezentrierungen sowie Verkippungen um die horizontale Achse auftreten [13, 15]. Allerdings sind die Ergebnisse im allgemeinen Fall mit 4 Freiheitsgraden (jeweils 2 für Dezentrierung und Verkippung) nicht mehr intuitiv und grafisch nicht mehr geschlossen darzustellen oder interpretierbar. Die horizontale Richtung für die Dezentrierung und die Verkippung um die vertikale Achse wurden in dieser Arbeit ausgewählt, da das Auge in horizontale Richtung wegen der Verkippung der Sehachse ohnehin keine Symmetrie aufweist. Aus den Ergebnissen kann direkt abgelesen werden, dass bei der Kataraktoperation mit Implantation einer Kunstlinse immer dann, wenn die Kunstlinse sich mit ihrer Haptikebene dezentriert oder verkippt gegenüber der Äquatorebene der natürlichen Augenlinse im Auge positioniert, ein Refraktionsfehler zu erwarten ist. Dieser Refraktionsfehler äußert sich zum einen in einer Abweichung der erreichten Refraktion (sphärisches Äquivalent, hier dargestellt über den Defokus) von der Zielrefraktion, aber auch in der Induktion eines Astigmatismus, der durch den keratometrisch, topografisch oder tomografisch gemessenen Hornhautastigmatismus alleine nicht erklärbar ist. Sehr viel wichtiger ist jedoch, dass neben den korrigierbaren Refraktionsfehlern Defokus und Astigmatismus zusätzlich mit der Coma ein wesentlicher Abbildungsfehler auftritt, der mit klassischen Korrekturverfahren wie Brille oder Kontaktlinse nicht korrigierbar ist. Die Abb. 4 zeigt, wenn auch nur für eine kleine Auswahl an 9 von 121 verschiedenen Szenarien für die Dezentrierung und Verkippung, welche Kombinationen im phaken Auge (Abb. 4a) und im pseudophaken Auge exemplarisch für die Kunstlinse des Typs Z9000 (Abb. 4b) mehr oder weniger von der Verschlechterung der Abbildungsqualität betroffen sind. So sieht man, dass das phake Liou-Brennan-Modellauge auch bei rotationssymmetrischen Hornhaut- und Linsengrenzflächen einen geringen Astigmatismus gegen die Regel (bei 90°) aufweist (Abb. 4a Mitte) und mit Ausnahme der Kombination aus Dezentrierung von −0,6 mm und 6° Verkippung (Bild links oben) die horizontale Coma die Abbildung dominiert. Ist das hier für die Simulation verwendete Kunstlinsenmodell (Z9000) perfekt nach der natürlichen Linse im Liou-Brennan-Augenmodell ausgerichtet, wird kein Astigmatismus beobachtet (Abb. 4b Mitte). Allerdings sind die anderen 8 Szenarien mit Dezentrierung und/oder Verkippung – auch die im Abschnitt vorher für das phake Auge hervorgehobene Kombination aus Dezentrierung von −0,6 mm und 6° Verkippung (Bild links oben) in abgeschwächter Form – durch die horizontale Coma in der Abbildung gekennzeichnet. Zum Vergleich wurden die entsprechenden Verhältnisse mit einer equikonvexen sphärischen Linse Kunstlinse untersucht. Die Linse wurde so modelliert, dass sie die gleichen Materialeigenschaften und den gleichen Brechwert (21 dpt) wie die vorher beschriebene Z9000 aufweist. Die Abb. 5 zeigt die Punktbildverwaschungsfunktion dieser sphärischen Linse. Bei einer Positionierung im Auge entsprechend der Vorgabe des Liou-Brennan-Modellauges (Dezentrierung und Verkippung jeweils 0, mittlere Grafik) weist das Auge eine deutliche sphärische Aberration auf, trotzdem sind etwa 80 % der Lichtenergie im zentralen Spot gebündelt. Der Spotdurchmesser ist hier mit etwa 16 µm deutlich größer im Vergleich zum phaken Auge oder dem pseudophaken Auge mit der Z9000, und die sphärische Aberration kommt auch in den anderen 8 Szenarien deutlich zum Tragen. Allerdings ist die horizontale Coma bei den 8 Szenarien mit Dezentrierung und/oder Verkippung ungleich 0 nicht so dominant im Vergleich zum pseudophaken Auge mit einer Z9000 (Abb. 4b).

Zum Schluss sei angemerkt, dass die hier verwendete Dezentrierung und Verkippung Extremwerte darstellen, die außerhalb der typischerweise gemessenen Werte liegen. Für die Dezentrierung von Kunstlinsen werden in der Literatur ein Mittelwert von ca. 0,3–0,4 mm angegeben sowie eine Dezentrierung von 3–5° [4], wobei hier zu beachten gilt, dass sich die Referenzachsen für die Angaben meist fundamental unterscheiden, sodass ein direkter Vergleich der einzelnen Studien nur schwer möglich ist. Legt man jedoch diese mittleren Werte als Messlatte zugrunde, so liegen die Werte für Defokus und Astigmatismus bei weniger 0,5 dpt und somit im Bereich der Zielgenauigkeit gängiger Berechnungsmethoden für Kunstlinsen. Somit können unvermeidbare Dezentrierung/Verkippung neben den Messunsicherheiten der Biometrie und der zulässigen Fertigungstoleranz für Kunstlinsen ursächlich sein für die Limitierung der Vorhersagegenauigkeit moderner Kataraktchirurgie.

## Fazit für die Praxis

Dezentrierung und Verkippung können im phaken wie auch im pseudophaken Auge in Kombination auftreten und die Abbildungsqualität deutlich beeinträchtigen. Ausgehend von einem modernen schematischen Modellauge von Liou-Brennan, wurde hier in einer optischen Simulation gezeigt, welcher Defokus und Astigmatismus durch eine Dezentrierung in horizontale Richtung und Verkippung um die vertikale Achse auftreten und wie ggf. eine verringerte Abbildungsqualität oder ein Astigmatismus im phaken oder pseudophaken Auge zu erklären ist, der auf der Basis keratometrischer, topografischer oder tomografischer Messdaten nicht von der Hornhaut herrührt.
